# Saliva‐derived cfDNA is applicable for *EGFR* mutation detection but not for quantitation analysis in non‐small cell lung cancer

**DOI:** 10.1111/1759-7714.13178

**Published:** 2019-08-23

**Authors:** Shanshan Ding, Xingguo Song, Xinran Geng, Lele Liu, Hongxin Ma, Xiujuan Wang, Ling Wei, Li Xie, Xianrang Song

**Affiliations:** ^1^ Department of Clinical Laboratory Shandong Cancer Hospital affiliated to Shandong University Jinan China; ^2^ Department of Clinical Laboratory, Shandong Cancer Hospital and Institute Shandong First Medical University and Shandong Academy of Medical Sciences Jinan China; ^3^ Shandong Provincial Key Laboratory of Radiation Oncology, Shandong Cancer Hospital and Institute Shandong First Medical University and Shandong Academy of Medical Sciences Jinan China; ^4^ Department of Clinical Laboratory, Maternity & Child Care Center of Dezhou Dezhou China; ^5^ School of Medicine and Life Sciences University of Jinan, Shandong Academy of Medicine Science Jinan China

**Keywords:** ddPCR, *EGFR* mutation detection, NSCLC, *s*cfDNA

## Abstract

**Background:**

Both quantitative and qualitative aspects of plasma cell‐free DNA (plasma cfDNA, pcfDNA) have been well‐studied as potential biomarkers in non‐small cell lung cancer (NSCLC). Accumulating evidence has proven that saliva also has the potential for the detection and analysis of circulating free DNA (saliva cfDNA, scfDNA).

**Methods:**

In the current study, we aimed to explore the potential application of scfDNA in NSCLC diagnostics and consistency of epidermal growth factor receptor (*EGFR*) mutation detection in paired pcfDNA and scfDNA using droplet digital PCR (ddPCR) and analyze the relationship between *EGFR* mutations and clinical treatment response.

**Results:**

In the quantitative cohort study, scfDNA concentration in NSCLC patients was no different from that in healthy donors, or in benign patients. ScfDNA concentration was significantly lower than pcfDNA concentration, yet they were not statistically significant in relevance (Spearman's rank correlation *r* = −0.123, *P* = 0.269). In the qualitative cohort study, the overall concordance rate of *EGFR* mutations between pcfDNA and scfDNA was 83.78% (31 of 37; k = 0.602; *P* < 0.001). *EGFR* mutation detection in paired pcfDNA and scfDNA was significantly correlated with the clinical treatment response (Spearman's rank correlation *r* = 0.664, *P* = 0.002).

**Conclusions:**

Our results demonstrated that saliva might not be the idea material for a cfDNA quantitative test, and scfDNA concentration is not applicable for NSCLC diagnostics. Conversely, scfDNA was capable of acting as the supplement for *EGFR* mutations due to the coincidence rate of *EGFR* mutation detection between scfDNA and pcfDNA.

## Introduction

Non‐small cell lung cancer (NSCLC) comprises 80% of all lung cancer cases and is the leading cause of cancer‐associated mortality worldwide.^1^ Most patients have local or distant metastasis at the time of diagnosis, whereas earlier tumor detection is associated with excellent survival.^2^ In recent years, circulating‐free DNA (cfDNA), released by both healthy and cancer cells into the bloodstream during apoptosis or necrosis, or by active secretion,^3^ has been proven to greatly impact molecular diagnostics of NSCLC patients due to simple, noninvasive access to genetic material detectable in plasma.^4^

The utility of plasma cell‐free DNA (pcfDNA) is not only to overcome the disadvantage of sampling limitations and heterogeneity in tissue biopsy, but also to reflect the status of genetic variation in real‐time.^5^ PcfDNA concentration has been shown to act as the biomarker in the diagnosis of NSCLC due to its ability to discriminate healthy subjects and NSCLC patients,^6^, ^7^, ^8^ as well as the predictor for disease progression since it is significantly related with treatment response.^9^, ^10^ Nowadays, the use of epidermal growth factor receptor (EGFR) tyrosine kinase inhibitors (TKIs) has become the standard therapeutic approach for NSCLC patients harboring sensitizing *EGFR* mutations, among which L858R mutation and 19 exon deletions (E19‐Dels) together account for approximately 90% of *EGFR*‐mutant tumors in the clinic.^11^ However, the majority of patients with *EGFR* mutations are found to be resistant (primary resistance) or gradually develop resistance (acquired resistance) after *EGFR*‐TKIs therapy, and T790M mutation is found in approximately 50%–60% of these cases.^12^ In mid‐2016, pcfDNA was approved by the U.S. Food and Drug Administration (FDA) for the identification of *EGFR* sensitizing mutations in basal setting (patients naive to any treatment) when tissue was not available or inadequate and in the progression setting for the identification of the *EGFR* T790M,^13^ suggesting the mutation detection in pcfDNA appears to be a promising and minimally invasive alternative to tumor biopsy for NSCLC patients.

Accumulating evidence has shown that saliva also has the potential for the detection and analysis of cfDNA. Saliva provides good‐quality genomic DNA which is comparable to blood as a template for genotyping.^14^, ^15^, ^16^ Saliva is produced by acinar cells in the salivary glands, which are highly permeable and surrounded by abundant capillaries, allowing molecules in the blood to exchange freely with those in adjacent salivary cells,^17^ thereby, most analytes detected in the blood are also found in saliva, indicating saliva should be considered as an ideal even better diagnostic fluid. It has been reported that the combined use of N‐α‐acetyltransferase 10 protein (Naa10p) and carcinoembryonic antigen (CEA) as tumor markers for oral squamous cell carcinoma (OSCC) in saliva were more sensitive than that in serum^18^; MiR‐21 in saliva was increased in colorectal cancer patients with a sensitivity of 97% and a specificity of 91%.^19^ Importantly, saliva can also be used for cfDNA (scfDNA) detection and analysis. *EGFR* mutations can be detected in the saliva of NSCLC patients using a novel core technology, called electric field‐induced release and measurement,^20^ processing the excellent detection efficiency with AUC (area under curve, after ROC analysis) of 0.96 and 0.94 for L858R and E19‐Dels, respectively. Despite these favorable attributes, the use of saliva as a diagnostic fluid seems to not yet have become a mainstream idea, mainly because the levels of most analytes in saliva which are quite different from those in blood are substantially diminished.^21^

In the current study, we aimed to explore the potential application of scfDNA at quantitative and qualitative levels in NSCLC. We studied the differences of scfDNA concentration between the case and control groups, then explored the consistency of *EGFR* mutation detection in paired pcfDNA and scfDNA and analyzed the relationship between *EGFR* mutations and clinical treatment response, providing novel insights of using saliva as a supplement to fluid biopsy materials.

## Methods

### Patients and healthy donors

The study included NSCLC patients admitted to the Department of Shandong Cancer Hospital Affiliated to Shandong University from June 2015 to August 2018. For the quantitative cohort study, 78 basal NSCLC patients naive to any anti‐tumor treatment, 15 patients with pulmonary benign disease and 26 healthy donors were recruited. For the qualitative cohort study, 40 NSCLC patients diagnosed with known *EGFR* mutations clinically using tumor tissue samples and six healthy donors were enrolled. No surgery was performed until collection of paired blood and saliva. All patients and healthy donors gave their informed consent for specimen collection and clinical information collection.

### Samples collection and cfDNA extraction

Peripheral blood samples were collected into EDTA tubes and centrifuged at 1900 ***g*** for 10 minutes at 4°C to separate the peripheral blood cells. The plasma was then further centrifuged at 16 000 ***g*** for 10 minutes at 4°C to pellet any remaining cells and was immediately stored at −80°C until DNA extraction.

Saliva was collected as reported previously.^22^ Briefly, all subjects were asked to refrain from eating, drinking, or oral hygiene for at least one hour prior to collection. They rinsed their mouths with water and used their tongues against the upper jaws to allow saliva to flow into an aseptic container. Participants were instructed not to cough or strongly expectorate in order to collect unstimulated saliva samples. Saliva was then centrifuged for 20 minutes at 300 × ***g*** to remove cells and another 20 minutes at 10 000 × ***g*** to remove cellular debris within one hour of collection, and was then stored at −80°C until DNA isolation.

PcfDNA and scfDNA were extracted using QIAamp Circulating Nucleic Acid Kit (Qiagen, Dusseldorf, Germany) according to the manufacturer's protocol. They were eluted into RNase free water, and the eluate reapplied onto the column for re‐elution. The final eluate was collected and stored at −20°C. Samples from paired plasma and saliva were always extracted together to avoid batch effects.

### Quantification of cfDNA by qPCR

Quantification of cfDNA was performed by SYBR Green based qPCR as described previously.^23^ Human‐specific primers were used for detection of human Long Interspersed Nuclear Element 1 (LINE1) retrotransposon, the primer sequences as follows: Forward, 5'‐GAAGTCAGTGTGGCGATTCC‐3′; and Reverse, 5'‐GGTTCCAAGTCTTTGCTATTGTG −3′. Serial dilutions from human leukocyte genomic DNA were used as calibrators for cfDNA quantification. For every independent experiment we made standard curves based on RNase free water to calculate cfDNA concentration of plasma and saliva, respectively.

### Cell line, gDNA extraction, droplet digital PCR

Droplet digital PCR (ddPCR) was established and analyzed using genomic DNA (gDNA) derived from the cell lines including human lung cancer‐derived cell lines HCC827 harboring *EGFR* E19‐Dels mutation, H1975 harboring *EGFR* T790M and L858R mutations and A549 harboring wild‐type *EGFR*, which were purchased from American Type Culture Collection (Manassas, VA, USA) and China Center for Type Culture Collection (Wuhan, China). All these cells were grown in Dulbecco's modified Eagle's medium (DMEM; Gibco, Invitrogen, Carlsbad, CA, USA) supplemented with 10% fetal bovine serum (Gibco, Invitrogen) and antibiotics (penicillin/streptomycin, 100 U/mL) at 37°C in 5% CO_2_.

Cellular gDNA was extracted using the genomic DNA extraction kit (Tiangen, Beijing, China) according to the manufacturer's protocol, and the gDNA concentration was determined by Nanodrop (TheromoFisher, Waltham, MA, USA) to calculate the amount of the sample.

DdPCR was performed on Bio‐Rad QX200 Droplet Digital PCR (Bio‐Rad Laboratories, Hercules, CA, USA) platform according to the manufacturer's protocol. Analysis of the ddPCR data was performed with QuantaSoft analysis software (version 1.7.4; Bio‐Rad Laboratories, Hercules, CA, USA) which accompanied the droplet reader. Sequences of primers and probes were purchased from Life Technologies (ThermoFisher) and are listed in Table 1.

**Table 1:** Sequence information of the primers and probes for the ddPCR assays

Mutation	Primer/probe ID	Sequence
E19‐Dels	E19‐F	5′‐ GTGAGAAAGTTAAAATTCCCGTC ‐ 3′
	E19‐R	5′ – TGGGCCTGAGGTTCAGA ‐ 3′
	E19‐Ref probe	5′ – FAM ‐ TGAGTTTCTGCTTTGCTGTGT‐MGB ‐ 3′
	E19‐Tar probe	5′ – VIC ‐ AGGAATTAAGAGAAGCAACAT – MGB ‐ 3′
T790M	E20‐F	5′ – GCCTGCTGGGCATCTGC ‐ 3′
	E20‐R	5′ – TCTTTGTGTTCCCGGACATAGTC ‐ 3′
	E20‐MUT probe	5′ – FAM – TCATCATGCAGCTCAT – MGB ‐ 3′
	E20‐WT probe	5′ – VIC – TCATCACGCAGCTCAT – MGB ‐ 3'
L858R	E21‐F	5′ ‐ CCGCAGCATGTCAAGATCAC ‐ 3'
	E21‐R	5′ – CCTCCTTCTGCATGGTATTCTTTCT ‐ 3'
	E21‐MUT probe	5′ – FAM – AGTTTGGCCCGCCCAA ‐ MGB ‐ 3'
	E21‐WT probe	5′ – VIC – AGTTTGGCCAGCCCAA – MGB ‐ 3'

ddPCR, droplet digital PCR; E19‐Dels, exon 19 deletions; F, forward primer; R, reverse primer; MUT, mutant allele; WT, wild‐type allele.

**Figure 1:**
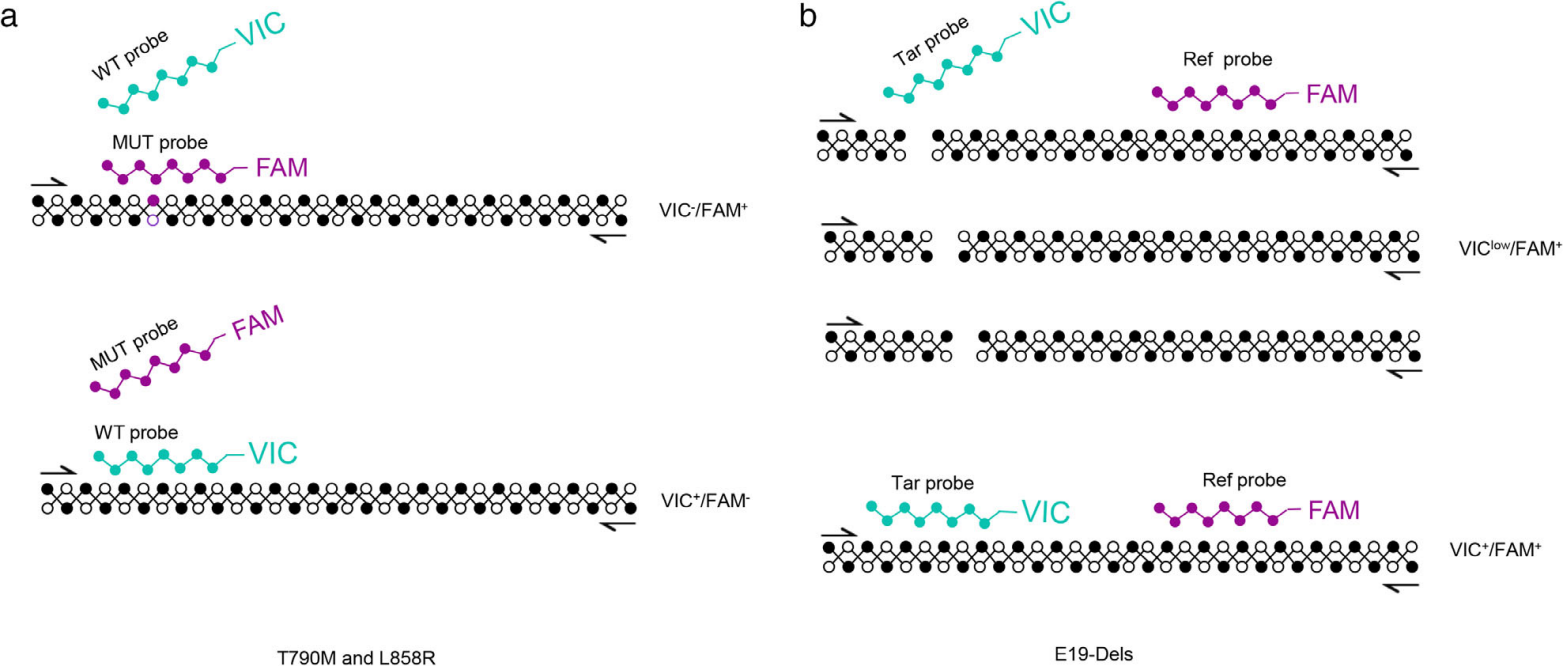
Design of the two assays for detection of *EGFR* E19‐Dels, T790M and L858R. For T790M and L858R assay (a), FAM^−^ and VIC^−^ labeled probes were designed to target the mutant and wild‐type EGFR alleles, respectively. For E19‐Del assay (b), The Ref probe was designed to target the nonmutated region, and the Tar probe was designed to target the mutation region. The Ref probe and Tar probe were labeled with FAM and VIC to detect the WT (VIC^+^/FAM^+^) and MUT (VIC^low^/FAM^+^). Our assay used a single probe covering the mutated region to detect all the mutations contained in hotspot regions. E19‐Dels, exon 19 deletions; MUT, mutant allele; WT, wild‐type allele.

The design principle of the ddPCR assays is shown in Fig 1. Briefly, for the L858R and T790M assays, two probes targeting a mutated region were labeled with FAM and VIC to detect the mutant and wild‐type *EGFR* allele with one nucleotide difference, respectively^11^; For E19‐Dels assay, as described previously,^24^ the Ref probe was designed to target the nonmutated region, and the Tar probe was designed to target the mutation region. WT molecules were double positive (VIC^+^/FAM^+^), and MUT molecules has low VIC signal (VIC^low^/FAM^+^). E19‐Dels assay was capable of detecting eight kinds of common exon19 deletions, as listed in Table 2.

**Table 2:** Eight kinds of common exon19 deletions detected by E19‐Dels assay

Mutation	Type	AA mutation	CDS mutation	Genomic coordinates	Mutation ID
E746_A750del	Deletion‐In frame	p.E746_A750delELREA	c.2235_2249del15	7:55174772… 55 174 786	COSM6223
E746_A750del	Deletion‐In frame	p.E746_A750delELREA	c.2236_2250del15	7:55174773… 55 174 787	COSM6225
L747_P753del	Complex‐deletion inframe	p.L747_P753 >S	c.2240_2257del18	7:55174777… 55 174 794	COSM12370
L747_T751del	Deletion‐In frame	p.L747_T751 delLREAT	c.2240_2254del15	7:55174777… 55 174 791	COSM12369
L747_A750del	Complex‐deletion inframe	p.L747_A750 > P	c.2239_2248TTAAGAGAAG > C	7:55174775… 55 174 785	COSM12422
E746_T751del	Complex‐deletion inframe	p.E746_T751 >A	c.2237_2251del	7:55174774… 55 174 788	COSM12678
L747_S752del	Deletion‐In frame	p.L747_S752 delLREATS	c.2239_2256del18	7:55174776… 55 174 793	COSM6255
L747_T751del	Deletion‐In frame	p.L747_T751 delLREAT	c.2238_2252del15	7:55174775… 55 174 789	COSM23571

### Statistical analysis

Statistical analysis was performed using SPSS 22.0 statistical software (SPSS, Chicago, IL, USA) and GraphPad Prism 6.0 (GraphPad Software, San Diego, CA, USA). For comparisons of non‐normal continuous variables, Wilcoxon test was used for two related samples and the Mann‐Whitney U‐test and the Kruskal‐Wallis H tests for two or more independent samples, respectively. Spearman correlation was used to compare the correlation between two variables. The *EGFR* status consistency between plasma and saliva was assessed by Kappa test. Data are presented as the median ± interquartile range (IQR) (range, minimum‐maximum). Significance was established at *P* < 0.05.

## Results

## Quantitative study cohort

### Study population

For the quantitative cohort study, a total of 78 basal NSCLC patients were recruited, three patients without definite diagnosis and seven pulmonary metastases from other cancers were ruled out, thereby, 68 NSCLC patients and 41 nontumor subjects including 15 patients with pulmonary benign disease and 26 healthy donors were subjected for the next research. Demographic information is listed in Table 3: age, gender, behavioral factors, pathological type, disease stage and tumor long diameter. Patients with pulmonary benign disease and healthy subjects matched for age, gender and risk factors were recruited as controls.

**Table 3:** Numbers and characteristics of cancer and noncancer donors

Characteristics	No. (%) of cancer and noncancer persons
**NSCLC**	68
Male	44 (64.7)
Age, median (min, max)	59 (26, 77)
**Behavioral factors**	
Smoking	38 (55.9)
Non‐smoking	30 (44.1)
Drinking	33 (48.5)
Nondrinking	35 (51.5)
**Pathological type**	
AC	39 (57.4)
SCC	28 (41.2)
Other	1 (1.5)
**Disease stage**	
I	37 (54.4)
II	6 (8.8)
III	16 (23.5)
IV	4 (5.9)
Unknown	5 (7.4)
**T category**	
T1	13 (19.1)
T2	38 (55.9)
T3	8 (11.8)
T4	3 (4.4)
Unknown	6 (8.8)
**Extracapsular spread (for N1–N3)**	
No	42 (61.8)
Yes	20 (29.4)
Unknown	6 (8.8)
**Distant metastasis**	
M0	59 (86.8)
M1	3 (4.4)
Unknown	6 (8.8)
**Tumor long diameter (cm)**	
≥2.7	23 (33.8)
<2.7	24 (35.3)
Unknown	21 (30.9)
**Patients with pulmonary benign diseases**	15
Male	11 (73.3)
Age, median (min, max)	58 (48,66)
**Pathological type**	
Pneumonia	3 (20)
Nodule	1 (6.7)
Phthisis	2 (13.3)
Benign tumor	4 (26.7)
Cyst	1 (6.7)
Lung space	3 (20)
Right lower lobe isolation	1 (6.7)
**Behavioral factors**	
Smoking	7 (46.7)
Non‐smoking	8 (53.3)
Drinking	6 (40)
Nondrinking	9 (60)
**Healthy donors**	26
Male	12 (46.2)
Age, median (min, max)	30 (24,62)
**Behavioral factors**	
Smoking	3 (11.5)
Non‐smoking	23 (88.5)
Drinking	3 (11.5)
Nondrinking	23 (88.5)

AC, adenocarcinoma; NSCLC, non‐small cell lung cancer; SCC, squamous cell carcinoma.

#### ScfDNA concentration is not applicable for NSCLC diagnostics

**Figure 2:**
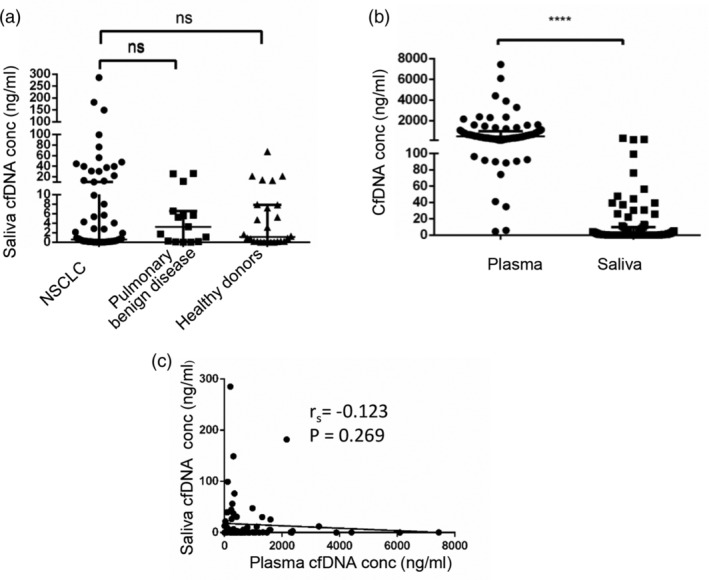
Quantification assays of scfDNA. (**a**) The differences of scfDNA level among NSCLC patients (*n* = 68), pulmonary benign disease patients (*n* = 15) and healthy donors (*n* = 26). (**b**) The differences of scfDNA and pcfDNA concentration in patients (*n* = 55 + 12). (**c**) The correlation of scfDNA and pcfDNA level in patients (*n* = 55 + 12). Spearman's correlation coefficient represented the degree of correlation. (Spearman's rank correlation, *r* = −0.123, *P* = 0.269). Solid lines represent median values. ****, *P* < 0.0001; NS, no significance; cfDNA, cell‐free DNA; scfDNA, saliva cfDNA; pcfDNA, plasma cfDNA; NSCLC, non‐small cell lung cancer; conc, concentration.

First, we determined whether scfDNA concentration was capable of acting as a biomarker for NSCLC diagnostics as outlined in the above mentioned cohort. The median concentration of scfDNA in healthy individuals was 1.11 (range, 0.01–67.63) ng/mL; in patients with pulmonary benign disease 3.26 (range, 0.029–26.30) ng/mL and in NSCLC patients 0.531 (range, 0.018–285.420) ng/mL, respectively. Unexpectedly, as shown in Fig 2a, scfDNA concentration in NSCLC patients was not different from that in healthy donors, or in benign patients. We also analyzed the relationship between scfDNA level and clinicopathological characteristics as listed in Table 4, and concluded that the age of the patient, their smoking or drinking history, pathological type, tumor size and disease stage was irrelevant to the results. Taken together, our data supported our conclusion that scfDNA concentration is not applicable in NSCLC diagnostics.

**Figure 3:**
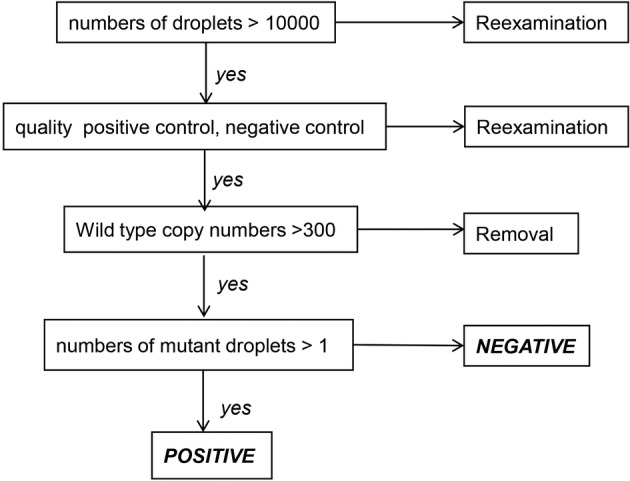
Establishment of *EGFR* mutations standard detection process. When ddPCR was performing, total numbers of droplets >10000, quality positive control and negative control, and wild type copy numbers >300 were required, otherwise, the samples were re‐examination or removal. It was defined as positive if numbers of mutant droplets >1, if not, it was defined as negative.

**Table 4:** Relationships between scfDNA concentration and characteristics

Characteristics	Numbers	*P*‐value
**Age**		0.7722
≤59	35	
>59	33	
**Gender**		0.9518
Male	44	
Female	24	
**Smoking history**		0.7427
Smoker	38	
Non‐smoker	30	
**Drinking history**		0.7397
Drinker	33	
Nondrinker	35	
**T category**		0.101*
T1	13	
T2	38	
T3	8	
T4	3	
**Nodal status**		0.0699
N0	42	
N1	20	
**Disease stage**		0.1004
I	37	
IIa‐IV	26	
**Pathological type**		0.7403
AC	39	
SCC	28	
**Tumor long diameter (cm)**		0.6016
≥2.7	23	
<2.7	24	

* Kruskal‐Wallis H test was used.

AC, adenocarcinoma; SCC, squamous cell carcinoma; scfDNA, saliva cfDNA.

Next, we analyzed the difference and correlation between scfDNA and pcfDNA concentration. As shown in Fig 2b, cfDNA concentration in saliva was much lower than that in plasma. Notably, the correlation between paired scfDNA and pcfDNA concentration was not prominent (Spearman's rank correlation, *r* = −0.123, *P* = 0.269; Fig 2c), and further supporting scfDNA concentration was not applicable for NSCLC diagnostics.

### Qualitative study cohort

#### Determination of the specificity and sensitivity of ddPCR assay

**Figure 4:**
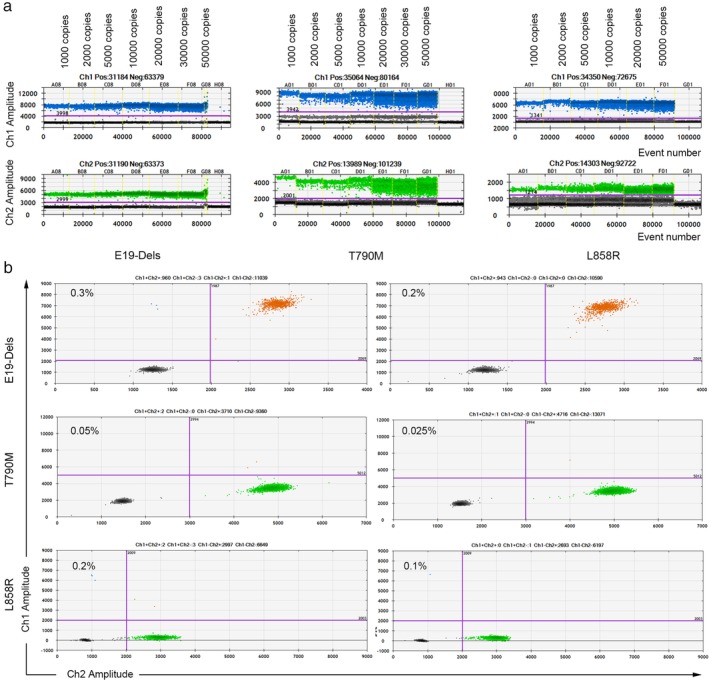
Specificity and sensitivity analysis for E19‐Dels, T790M and L858R methods. (**a**) For specificity analysis, the established ddPCRs were performed using related gDNA (HCC827 gDNA harboring *EGFR* E19‐Dels mutation, H1975 gDNA containing *EGFR* T790M and L858R double mutations) with different copies from 1000 to 50 000. (**b**) For sensitivity analysis, the established ddPCRs were performed using gradual diluted mutant gDNA with the wild‐type (A549 gDNA harboring wild‐type *EGFR*).

The ddPCR method was established as shown in “Methods” and followed the standard in Figure 3. To determine the specificity, the ddPCR assay were performed using related gDNA (cell lines HCC827 harboring *EGFR* E19‐Dels mutation, H1975 harboring *EGFR* T790M and L858R mutations and A549 harboring wild‐type *EGFR*) with different copies from 1000 to 50 000. As shown in Fig 4a, distinct separation between the positive and negative droplets demonstrated the excellent specificity of the established methods even gDNA amount reached 50 000 copies in the 1D Amplitude chart. Moreover, the sensitivity of ddPCR assay was also analyzed through testing serial dilutions of *EGFR* mutants by mixing DNA derived from the positive cell lines with wild‐type, and their sensitivity was 0.3%, 0.05%, 0.2%, respectively (Fig 4b).

#### Study population

**Figure 5:**
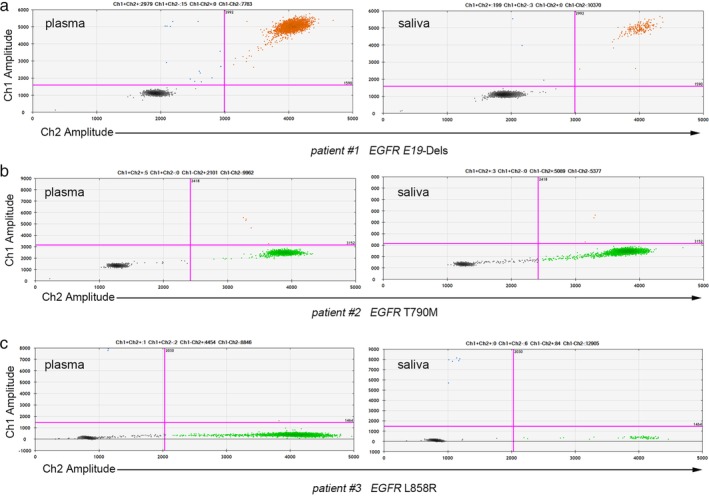
Two‐dimensional maps of *EGFR* mutation detection using ddPCR. DdPCR was performed via Bio‐Rad QX200TM Droplet Digital PCR system. Representative two‐dimensional maps for *EGFR* mutations including (**a**) E19‐Dels, (**b**) T790M and (**c**) L858R detection were displayed.

For the qualitative cohort study, paired scfDNA and pcfDNA samples were collected from 40 NSCLC patients diagnosed with indicated *EGFR* mutations clinically, as well as from six healthy donors,and subjected to ddPCR analysis. Representative two‐dimensional maps for *EGFR* mutations including E19‐Dels, T790M and L858R detection were displayed in Fig 5. At last, 13 paired samples were ruled out due to copy numbers of scfDNA <300, thereby 27 paired samples were enrolled in the research with full information (Table S1).

#### Consistency of *EGFR* mutation detection using scfDNA and pcfDNA

Next, we analyzed consistency of *EGFR* mutation detection in the above mentioned cohort using paired scfDNA and pcfDNA. As shown in Table 5, for the E19‐Dels ddPCR assay, 12 patients were detected, among which four patients were positive and five patients were negative in both plasma and saliva, whereas three patients were positive in plasma but undetectable in saliva. For the L858R ddPCR assay, 14 patients were detected, among which two patients were positive and nine patients were negative in both plasma and saliva, whereas three patients were positive in plasma, but undetectable in saliva. For the T790M ddPCR assay, only one patient was positive in the paired plasma and saliva samples. Healthy donors provided 10 paired blood and saliva samples, three of which were tested for L858R and T790M, respectively and four for E19‐Dels; the results were negative and coincident in paired plasma and saliva.

**Table 5:** Consistency of *EGFR* mutation detection in paired plasma and saliva

	Saliva		
	+	−	Total
**NSCLC patients**			
**Plasma**			
**E19‐Dels**			
+	4	3	7
‐	0	5	5
Total	4	8	12
**L858R**			
+	2	3	5
−	0	9	9
Total	2	12	14
**T790M**			
+	1	0	1
−	0	0	0
Total	1	0	1
**Healthy donors**			
**Plasma**			
E19‐Dels			
+	0	0	0
‐	0	4	4
Total	0	4	4
**L858R**			
+	0	0	0
−	0	3	3
Total	0	3	3
**T790M**			
+	0	0	0
−	0	3	3
Total	0	3	3

Collectively, seven paired NSCLC samples were positive, 24 paired samples including 14 NSCLC and 10 healthy were negative for *EGFR* mutations (E19‐Dels, T790M, L858R) in the 37 qualified paired plasma and saliva samples. However, six samples positive in pcfDNA, were not detected with *EGFR* mutations in the paired scfDNA. Collectively, the overall concordance rate between pcfDNA and scfDNA was 83.78% (31 of 37; k = 0.602; *P* < 0.001) (Table 6). Our data suggested saliva‐based qualitative testing was applicable for *EGFR* mutation detection in NSCLC, indicating saliva as a supplement to blood‐ and tissue‐based biopsy.

**Table 6:** *K*appa analysis for consistency of *EGFR* mutation detection in paired plasma and saliva samples

		Saliva				
		+	−	Total	*P*‐value	Kappa value
Plasma	+	7	6	13	<0.001	0.602
	−	0	24	24		
	Total	7	30	37		

#### Application of EGFR mutation detection in pcfDNA and scfDNA for clinical treatment response assessment

Finally, we analyzed the relationship between *EGFR* mutation status detected in pcfDNA and scfDNA and clinical treatment response in the 27 NSCLC patients whose tumors harbored common *EGFR* mutations which were confirmed by tissue biopsy. They underwent a series of clinical anticancer treatments, and 19 had clear efficacy evaluation (Table S1) according to RECIST guidelines.^25^ For eight patients with stable disease (SD), two were positive (DP) and five were negative (DN) for *EGFR* mutations in blood and saliva, whereas one was positive in plasma but undetectable in saliva (SP). For seven patients with progressive disease (PD), two were DP and five were SP. Four patients with partial response (PR) were all DN. In total, *EGFR* mutation detection in paired pcfDNA and scfDNA was significantly correlated with the clinical treatment response (Spearman's rank correlation, *r* = 0.664, *P* = 0.002; Fig S1).

## Discussion

In the current study, we explored the potential application of scfDNA in NSCLC diagnostics and consistency of *EGFR* mutation detection in paired plasma and saliva samples using ddPCR. The results demonstrated that saliva cfDNA is applicable for *EGFR* mutation detection but not for quantitation analysis in NSCLC.

We studied the relationship between scfDNA concentration and clinicopathological features of NSCLC patients, in which no significant differences were detected including age, gender, and pathological type, coincident with the studies on pcfDNA.^26^, ^27^ However, although previous studies had reported pcfDNA level acted as the biomarker in the diagnosis of NSCLC due to its ability to discriminate healthy subjects and NSCLC patients,^6^, ^7^, ^8^ our data demonstrated that scfDNA concentration was not applicable for NSCLC diagnostics. In fact, scfDNA originated from pcfDNA which originated from tumor tissue,^28^, ^29^ resulting in its low concentration, thus preventing scfDNA diagnostics in clinical practice.^30^ In addition, saliva viscosity might alter within a person and between individuals.^31^ Since saliva secretion is affected by various uncontrollable factors such as emotional and mental influences, even some participants are often unwilling or unable to actively participate in saliva expectoration,^32^ causing a large variation in scfDNA concentration, even in the same individual at different collection times. Another challenge is that saliva collecting, processing and testing methods are needed to standardize to eliminate the scientific validations.^32^, ^33^ In the current study, 68 NSCLC patients, 15 pulmonary benign disease patients and 26 healthy donors were enrolled, a tendency that scfDNA was elevated in NSCLC patient was observed, despite being of no statistical significance, thereby, we also would not deny the applicability of scfDNA for NSCLC diagnostics in an expanded cohort.

Owing to the development of hypersensitive techniques, the low concentration of analytes in saliva is no longer a limit.^30^ As an emerging platform, ddPCR distributed PCR reaction into discrete droplets, enabling the accurate detection and quantification of molecular targets, single molecule analysis by this manner is accurate, cost‐effective, and readily performed.^34^, ^35^ In our qualitative study, we performed ddPCR to detect and compare *EGFR* mutations in scfDNA and pcfDNA. The overall concordance rate between them was 83.78% (31 of 37; k = 0.602; *P* < 0.001) (Table 6), suggesting scfDNA would become a supplement for *EGFR* mutations beside plasma and tissue. Nevertheless, six patients whose *EGFR* mutations were positive in the pcfDNA, were not detected with *EGFR* mutations in the paired scfDNA. This might be attributed to the low scfDNA concentration and low mutations frequency as discussed above, since higher DNA input amounts could achieve associated increase in sensitivity and higher detection rate.^36^ Besides, 14 patients were still negative in the paired plasma and saliva samples, although 27 patients were previously diagnosed with *EGFR* mutations. This was because patients underwent TKIs targeted, radiation or other related treatments after pathological diagnosis and before collection of plasma and saliva samples, which reduced *EGFR* mutation frequency in NSCLC patients.^37^, ^38^

More importantly, our data revealed a potential correlation of *EGFR* mutation detection to response to clinical treatment, but conclusion was limited due to the small sample (only 19 patients involved with clear efficacy evaluation) and single arm study design. However, our data demonstrated a clear value to predict therapeutic effect of *EGFR* mutation detection in paired pcfDNA and scfDNA in NSCLC. Patients with *EGFR* mutations positive both in paired pcfDNA and scfDNA had worse clinical treatment response, which strengthened the potential clinical implication in monitoring treatment effect.

Nevertheless, although scfDNA concentration appears not to be applicable for NSCLC diagnostics because of its extremely low content, salivary circulating free miRNAs have recently become an emerging field for diagnosing or monitoring cancer.^39^ Due to its short length and resistance to RNases degradation, salivary miRNA could be exchanged more freely between plasma and saliva, thereby composing over 50% of total salivary RNA. It has been reported salivary miRNAs acted as biomarkers for oral cancer head and neck squamous cell carcinomas, esophageal cancer and even gastric cancer,^40^, ^41^, ^42^, ^43^ implying its potential role in the early diagnosis for NSCLC patients.

In conclusion, saliva might not be the ideal material for a cfDNA quantitative test, and scfDNA concentration not applicable for NSCLC diagnostics. However, scfDNA was capable of acting as the supplement for *EGFR* mutations beside plasma and tissue due to the coincidence rate of *EGFR* mutation detection between scfDNA and pcfDNA.

## Disclosure

No authors report any conflict of interest.

## Supporting information


**Figure S1** The correlation between clinical response and *EGFR* mutations detection in paired pcfDNA and scfDNA. Three‐wire table (a) and Bar plot (b) illustrated *EGFR* mutations detection results in patients with different clinical response. DP represented patients were positive for *EGFR* mutations in both plasma and saliva; DN showed that patients were negative for *EGFR* mutations in both plasma and saliva; SP indicated that patients were positive in plasma but not in saliva. Spearman's correlation coefficient represented the degree of correlation (Spearman's rank correlation *r* = 0.664, *P* = 0.002). SD, stable disease; PD, progressive disease; PR, partial response; DP, double positive; DN, double negative; SP, single positive.

**Table S1** Summary of patient demographics and mutations detection results by ddPCR.
