# Supplementary figures and images for: Saliva‐derived cfDNA is applicable for EGFR mutation detection but not for quantitation analysis in non‐small cell lung cancer
Source: Thorac Cancer. 2019 Aug 23;10(10):1973–83. doi: 10.1111/1759-7714.13178 (PMC6775000; doi:10.1111/1759-7714.13178)

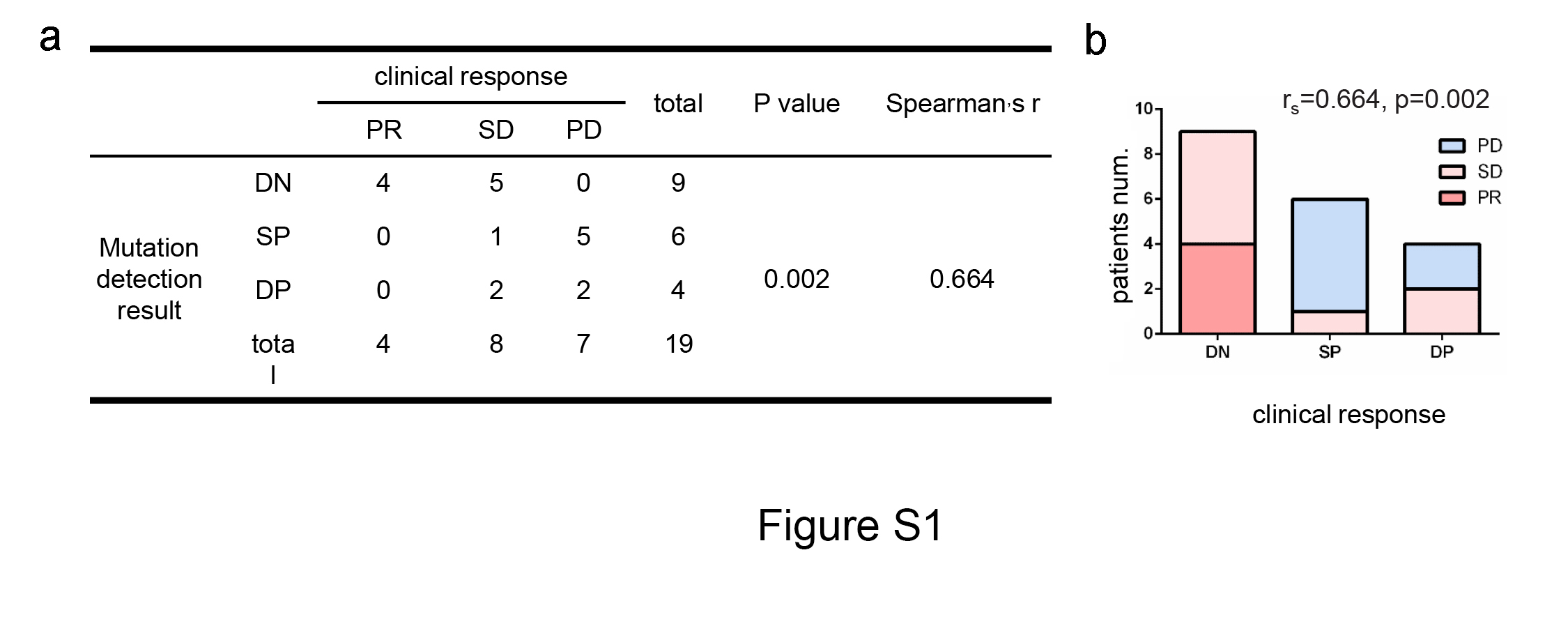

Supplement: Supplementary file 1 — Figure S1 The correlation between clinical response and EGFR mutations detection in paired pcfDNA and scfDNA. Three‐wire table (a) and Bar plot (b) illustrated EGFR mutations detection results in patients with different clinical response. DP represented patients were positive for EGFR mutations in both plasma and saliva; DN showed that patients were negative for EGFR mutations in both plasma and saliva; SP indicated that patients were positive in plasma but not in saliva. Spearman's correlation coefficient represented the degree of correlation (Spearman's rank correlation r = 0.664, P = 0.002). SD, stable disease; PD, progressive disease; PR, partial response; DP, double positive; DN, double negative; SP, single positive. [file TCA-10-1973-s001.jpg]
